# Optimized high gradient magnetic separation for isolation of Plasmodium-infected red blood cells

**DOI:** 10.1186/1475-2875-9-38

**Published:** 2010-02-02

**Authors:** Sebastian C Bhakdi, Annette Ottinger, Sangdao Somsri, Panudda Sratongno, Peeranad Pannadaporn, Pattamawan Chimma, Prida Malasit, Kovit Pattanapanyasat, Hartmut PH Neumann

**Affiliations:** 1 Department of Pathobiology, Faculty of Science, Mahidol University, Rama VI Road, Bangkok 10400, Thailand; 2 X-Zell Biotech Ltd., Thailand Science Park, Pathumthani, Thailand; 3 Division of Instruments for Research, Office for Research and Development, Faculty of Medicine Siriraj Hospital, Mahidol University, Bangkok 10700, Thailand; 4 Institute of Medical Microbiology and Hygiene, Johannes Gutenberg University, 55101 Mainz, Germany; 5 Department of Parasitology, Biomedical Parasitology Unit, Pasteur Institute, Paris, France; 6 Division of Medical Molecular Biology, Office for Research and Development, Faculty of Medicine Siriraj Hospital, Mahidol University, Bangkok 10700, Thailand; 7 Preventive Medicine Unit, University Medical Center, Albert-Ludwigs-University of Freiburg, 79104 Freiburg, Germany

## Abstract

**Background:**

Highly purified infected red blood cells (irbc), or highly synchronized parasite cultures, are regularly required in malaria research. Conventional isolation and synchronization rely on density and osmotic fragility of irbc, respectively. High gradient magnetic separation (HGMS) offers an alternative based on intrinsic magnetic properties of irbc, avoiding exposure to chemicals and osmotic stress. Successful HGMS concentration in malaria research was previously reported using polymer coated columns, while HGMS depletion has not been described yet. This study presents a new approach to both HGMS concentration and depletion in malaria research, rendering polymer coating unnecessary.

**Methods:**

A dipole magnet generating a strong homogenous field was custom assembled. Polypropylene syringes were fitted with one-way stopcocks and filled with stainless steel wool. Rbc from *Plasmodium falciparum *cultures were resuspended in density and viscosity optimized HGMS buffers and HGMS processed. Purification and depletion results were analysed by flow cytometer and light microscopy. Viability was evaluated by calculating the infection rate after re-culturing of isolates.

**Results:**

In HGMS concentration, purity of irbc isolates from asynchronous cultures consistently ranged from 94.8% to 98.4% (mean 95.7%). With further optimization, over 90% of isolated irbc contained segmented schizonts. Processing time was less than 45 min. Reinfection rates ranged from 21.0% to 56.4%. In HGMS depletion, results were comparable to treatment with sorbitol, as demonstrated by essentially identical development of cultures.

**Conclusion:**

The novel HGMS concentration procedure achieves high purities of segmented stage irbc from standard asynchronous cultures, and is the first HGMS depletion alternative to sorbitol lysis. It represents a simple and highly efficient alternative to conventional irbc concentration and synchronization methods.

## Background

Malaria remains one of the world's major health burdens. With 2.5 billion people at risk it affects an estimated 500 million people annually, causing one to three million deaths, the majority of which occurs in children under five years of age. Improved methods facilitating research in the field are urgently needed [1,2].

Isolation of infected red blood cells (irbc) is a crucial step in basic and applied malaria research. For the past three decades, isolation has been performed mostly by Percoll^® ^density gradient separation, exploiting the fact that density of irbc decreases with parasite maturation [3]. A further refinement of this method are hypertonic, discontinuous Percoll^®^-sorbitol gradients, where particular fractions of irbc can be obtained. Hypertonicity causes cell shrinkage of rbc, while irbc swell back due to an influx of sorbitol through new permeability pathways. This increases the density gaps between the different developmental stages and allows better separation than in pure Percoll^® ^gradients [4]. Purification results, however, depend on a variety of factors, including individual research experience. Gelatin sedimentation is used as an alternative concentration method, however, it is useful only for parasite strains exhibiting knobs [5].

Frequently, not only highly purified but also stage-synchronized parasite cultures and isolates are required. Synchronization of cultures is performed by isotonic sorbitol lysis of late-stage irbc, as described 30 years ago [6]. Time-consuming synchronization cycles by repeated sorbitol lysis and/or Percoll^® ^isolation are required to obtain synchronized and pure irbc suitable for downstream applications [6-8]. While sorbitol selectively lyses late-stage irbc, it imposes sub-lytic osmotic stress in younger stage irbc and likely enters these cells [9]. Whether or not exposure of irbc to synthetic chemicals and osmotic stress, respectively, has unwanted consequences on parasites remains an open question.

In principle, high gradient magnetic separation (HGMS) offers a way to concentrate or deplete malaria irbc from suspensions, relying solely on their intrinsic magnetic properties. Particularly late-stage irbc are known to behave as paramagnetic particles [10]. In a paramagnetic particle, magnetic poles are induced only when exposing the particle to a magnetic field, the removal of which leads to immediate de-magnetization. Due to the very small distance separating the particle's respective north- and south-poles, very high magnetic field gradients are required to create a net magnetic force, which is able to attract or repel the particle. Such gradients are generated by placing thin filamentous or spherous ferromagnetic material as a matrix into a strong homogenous magnetic field, which is usually provided by rare-earth dipole magnets or electromagnets. With this technology, magnetic gradients up to 100 Tesla/cm can be created at the surface of the matrix [11].

Paramagnetism in malaria irbc results from the hemoglobin catabolism of intra-erythrocytic malaria parasites. Free haem as a toxic by-product is de-toxified by polymerization and by oxidation of the molecule's central iron atom [12]. Oxidized iron [Fe^+3^] carries five unpaired electrons in its d-orbitals, rendering the molecule and the resulting polymer paramagnetic [10,13,14]. Deposition and accumulation of polymerized haem (haemozoin) in the parasite's food vacuole result in a continuously increasing magnetic susceptibility of the irbc [10].

Successful, but not highly efficient HGMS of late-stage irbc from malaria cultures was first described in 1981 [14]. Later, commercially available, polymer coated HGMS columns were shown to offer improved results [15-18]. Recently, successful synchronization was demonstrated with polymer coated HGMS columns. However, sorbitol-pretreatment of Plasmodium cultures was essential [19]. Generally, the use of HGMS in malaria research is still hampered by limited column capacity, inconsistent separation purities and high costs.

Firstly, this study presents a buffer-optimized HGMS concentration system for irbc, as an alternative to Percoll^® ^density gradient separation. Secondly, a HGMS-BSA gradient system is introduced for HGMS depletion of late-stage irbc, as an alternative to Sorbitol lysis.

Buffer-optimized HGMS was developed by identifying the three key variable parameters relevant to HGMS for cell separation. Briefly, these are derived as follows:

Particles in an HGMS device can be captured only if(1)

where  is the magnetic force,  the drag force and  the gravitational force acting on the particle.

The gravitational force is defined by Newton's law:(2)

where *m *is the mass of the particle and  the gravitational acceleration.

The drag force is defined by Stoke's law:(3)

where *η *is the viscosity of the carrier fluid, *r *the radius and  the velocity of the particle. While the radius of the rbc is constant and the viscosity of the carrier fluid depends on its defined chemical properties, the velocity of the particle is further determined by two vectors:(4)

where  is the velocity of the carrier fluid and  the sedimentation velocity of the particle. Here, while the velocity of the carrier fluid can be easily adjusted, the sedimentation velocity deserves further discussion:(5)

where *d*_*p *_is the density of the particle, *d*_*f *_the density of the carrier fluid,  the gravitational acceleration, *r *the radius of the particle and η the viscosity of the carrier fluid [20].

Analysing equation (1)-(5), it becomes obvious that apart from the constant intrinsic parameters of the particle, there are only four extrinsic parameters governing the HGMS system. These are a) the gravitational acceleration, b) the velocity of the carrier fluid, c) the viscosity of the carrier fluid, and d) the density of the carrier fluid.

It was hypothesized that in an ideal buffer-optimized HGMS system, the density of the carrier fluid can be adjusted to the density of rbc, which would render the influence of the gravitational acceleration on the particle negligible, setting the particle sedimentation velocity close to zero. In this case, particle velocity would be perfectly controllable solely by adjusting the velocity of the carrier fluid. Additionally, the density-concurrent increase in viscosity *η *would favourably support laminar flow conditions in the column.

To test this hypothesis, commonly available raw materials were employed to perform selective, buffer-optimized HGMS of *P. falciparum *late-stage irbc from asynchronous, standard routine malaria cultures, avoiding exposure to anything but incomplete malaria culture medium (RPMI 1640) or isotonic sucrose solution, substituted with either BSA or gelatine, respectively. A concentration protocol was developed as an alternative to Percoll^® ^separation and HGMS with polymer-coated columns. We show that with further refinement, optimized HGMS results in unprecedented purities of segmented-stage irbc from standard asynchronous cultures.

The second protocol demonstrates efficient depletion of late-stage irbc from Plasmodium cultures. Here, a BSA density gradient was introduced within the HGMS separation column, which prevented uncontrolled sedimentation of rbc during incubation within the matrix of the column. It is demonstrated that HGMS depletion alone, without HGMS concentration, can be used as an alternative to Sorbitol lysis for culture synchronization, potentially further widening the application of HGMS in malaria reserach.

## Methods

### Parasite strains

The *P. falciparum *TM 267 laboratory strain was kindly provided by the Hospital of Tropical Diseases, Faculty of Tropical Medicine, Mahidol University, Bangkok. Parasites were passaged in human rbc blood group O in RPMI 1640 medium containing HEPES 5.94 g/l, glucose 1 g/l, hypoxanthine 50 mg/l, 5% sodium bicarbonate and 10% human AB serum at 5% haematocrit in an incubator under 5% CO_2 _atmosphere at 37°C.

### Reagents

Hydroethidine (HE) and acridine orange (AO) were purchased from Invitrogen, Molecular Probes (Eugene, OR). Sheath fluid for flow cytometry was from Becton Dickinson Bioscience (BDB) (San Jose, CA), all other reagents were from Sigma-Aldrich (St. Louis, MO). Human blood for parasite cultures was from volunteers in our laboratory and human AB serum from the Blood Bank of Siriraj Hospital, Bangkok.

### Depletion of late-stage irbc by sorbitol lysis

Sorbitol lysis was performed as described previously [6]. Briefly, 50 μl of packed cells from *P. falciparum *culture where re-suspended in 1 ml of aqueous 5% D-sorbitol solution and incubated at 37°C for 15 min, followed by centrifugation at 900 g for 5 min. The supernatant and the dark layer of free late-stage parasites from lysed irbc were discarded. The remaining cells were washed once in 10 ml RPMI 1640 and re-cultured.

### HGMS apparatus, columns and buffer

A dipole magnet was constructed as described previously [11,14]. Briefly, two 50 × 30 × 12 mm neodymium magnets (Neotexx, Berlin, Germany) were mounted on a custom made, U-shaped iron-yoke, generating a uniform field strength of a minimum of 0.7 Tesla between the poles. For HGMS concentration, a 3 ml polypropylene syringe (Terumo, Ayutthaya, Thailand) was filled with 1 g of stainless steel wool of 30-50 μm diameter (Oscar Weil, Lahr, Germany) and was fitted with a one-way stopcock (Value Plastics, Fort Collins, CO). For HGMS depletion, a 5 ml syringe was filled with 4 g of stainless steel wool. HGMS buffers were isotonic sucrose with 0.75% gelatin (concentration buffer) or RPMI 1640 with 0.2% and 1% BSA (depletion buffers) (all chemicals from Sigma-Aldrich, St. Louis, MO). Columns were autoclaved for re-culturing experiments.

### HGMS protocol for concentration of irbc

For isolation of late-stage irbc, the column was filled with concentration buffer in upright position to evacuate air by upward displacement. After removing remaining air bubbles by gentle finger tapping, a 20G/1-inch injection needle was connected to the stopcock. The column was placed between the poles of the dipole magnet and equilibrated for 5 min. Different volumes of cultures containing malaria irbc were centrifuged at 800 g for 5 min, washed in RPMI 1640, resuspended in concentration buffer at a final haematocrit of 1-5%, and oxygenated against ambient air for 5 min. After oxygenation of rbc was completed, the stopcock of the HGMS column was opened and the rbc suspension applied. Without interrupting the flow, the column was rinsed with 45 ml of concentration buffer. After this, the stopcock was closed and the column was removed from the magnet. The needle was removed from the stopcock and the column retrogradely flushed with 12 ml of PBS or isotonic sucrose solution. The eluate containing isolated late-stage irbc was collected, centrifuged at 800 g for 5 min, washed once with RPMI 1640 analysed and re-cultured.

### HGMS protocol for depletion of irbc

For depletion of late-stage irbc, the column was filled with RPMI 1640 containing 1% BSA in upright position to evacuate air by upward displacement. After removing remaining air bubbles by gentle finger tapping, a 23G/1 inch injection needle was connected to the stopcock. The column was placed between the poles of the dipole magnet and equilibrated for 5 min. Different volumes of cultures containing malaria irbc were centrifuged at 800 g for 5 min, washed in RPMI 1640, resuspended in RPMI 1640 containing 0.2% BSA at a final haematocrit of 3.3%, and oxygenated against ambient air for 5 min. The difference in BSA concentration created a density gradient sufficient to stop uncontrolled sedimentation of rbc within the matrix. After oxygenation of rbc was completed, the stopcock was opened and the rbc suspension applied to the top of the column. When the rbc suspension had completely entered the matrix, the flow was stopped by closing the stopcock. The column was left between the poles of the dipole magnet for 30 min, which in pilot experiments was determined to be the optimal incubation time. After incubation, the column was rinsed with 8 ml of RPMI 1640/0.2% BSA and the flow-through collected, centrifuged at 800 g for 5 min, washed once and re-cultured.

### Analysis of purity and synchronization

Purity of isolates and synchronization of re-culture was analysed by Giemsa stained blood smears and by flow cytometer (BD FacsCalibur). For microscopic counting, stages were defined as follows:

• ring-stage trophozoites: clearly visible ring-shaped cytoplasm, and one to two chromatin dots

• young trophozoites: small solid cytoplasm (occupying the lesser fraction of the erythrocyte's cytoplasm), one nucleus and little, light-brown pigment

• late trophozoites: large solid cytoplasm (occupying the larger fraction of the erythrocyte's cytoplasm), one nucleus and solid, dark-brown pigment

• schizonts: parasites with two or more nuclei

For flow cytometry, staining was performed with HE or AO, as described previously. For the first, 50 μl of *P. falciparum *culture with a haematocrit of 5% were mixed with HE at a final concentration of 5 μg/ml in a final volume of 55 μl, followed by incubation in the dark at 37°C for 30 min. 500 μl of PBS were added and data acquired in the flow cytometer. Logarithmic orange fluorescence was observed through a 585/42 nm band pass filter in the FL-2 channel and compensation set to 0 [21]. For the second, 5 μl of *P. falciparum *culture with a hematocrit of 5% were mixed with AO at a final concentration of 0.5 μg/ml in a final volume of 200 μl. The mixture was incubated in the dark at room temperature for 5 min. 500 μl of PBS were added and data acquired in the flow cytometer. Logarithmic green was detected using a 530/30 nm bandpass filter and logarithmic red fluorescence using a 630 nm longpass filter in the FL-1 channel and FL-3 channel, respectively. Compensation was set to 0 [22].

## Results

### Concentration of late-stage irbc from cultures by buffer-optimized HGMS

**Figure 1:**
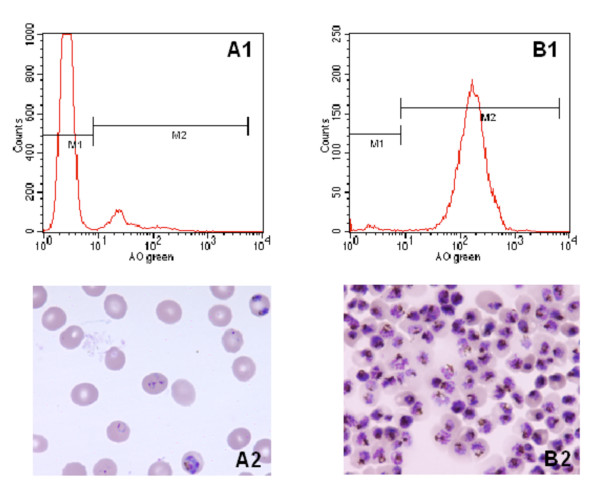
**Morphology and corresponding flow cytometric analysis of *P. falciparum *irbc before and after buffer optimized HGMS**. 5 × 10^8 ^rbc from culture were applied to a HGMS column as described above. Parasitemia was measured by flow-cytometer after staining with Acridine Orange (100,000 events counted). Histograms (A1 and B1) and corresponding blood smears (A2 and B2) of one representative experiment are shown. M1 non infected rbc, M2 infected rbc. A1 and A2: Culture with a total parasitemia of 10.9%. B1 and B2: Result of buffer optimized HGMS of irbc from the culture: irbc containing late trophzoites and schizonts are enriched to a concentration of 98.4%.

For analysis of purity and yield of buffer-optimized HGMS, non-synchronized cultures were grown to total parasitaemias of 5-10%. To examine the yield of isolates, a total number of 5 × 10^8 ^of rbc was applied to the column. The average yield was 12.1 × 10^6 ^irbc (S.D. = 2.6 × 10^6^) in six experiments, independent of culture parasitaemia within the range mentioned above. Purities of irbc ranged from 94.23 to 98.26% (Table 1). Morphology of isolates and corresponding analysis by flow cytometry of a representative experiment are shown in figure 1.

**Table 1:** Yield and purity of isolates in six independent experiments

Experiment no.	Yield [total no. of irbc purified]	Purity irbc/total cells [%]
1	12,787,500	98.46

2	16,937,500	94.23

3	11,287,500	95.42

4	9,962,500	94.87

5	11,462,500	96.43

6	10,325,000	95.05

**Mean**	**12,127,083**	**95.74**

S.D.	2,600,000	1.38

5 × 10^8 ^of rbc from cultures with 5-10% parasitemia were applied to the HGMS column. Experiments were performed from different cultures on different days. Purity was determined by flow cytometry, as described in material and methods.

**Figure 2:**
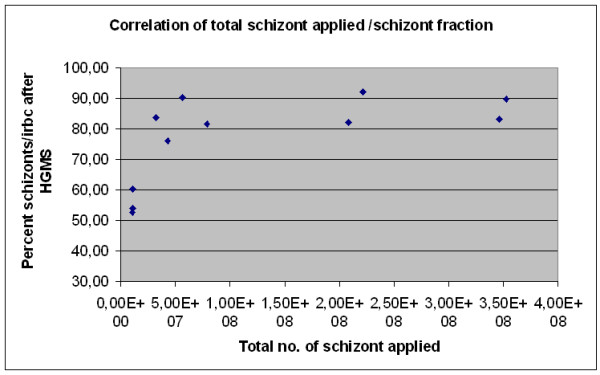
**Schizont-irbc fraction of isolates in eleven independent experiments**. Total numbers of 2.5 × 10^8 ^to 3 × 10^9 ^rbc from cultures with 5-15% total parasitemia were applied to the HGMS column. Increasing the total number or schizont-irbc applied to the column resulted in higher fractions of schizont-irbc in total irbc. Total irbc purities ranged from 93.20 to 98.40% (Mean 95.69%, S.D. 1.52).

**Figure 3:**
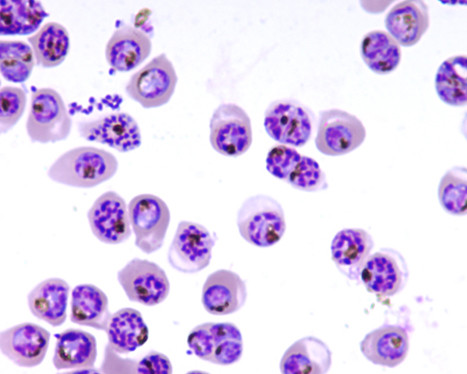
**Isolation of segmented stage irbc by increased irbc loading of buffer optimized HGMS columns**. In this representative experiment, 3 × 10^9 ^rbc from an asynchronized culture containing app. 3% schizont irbc were applied to a HGMS column. Over 90% of segmented stage irbc are obtained, total irbc purity is >98%. Differential parasitemias were counted microscopically (1000 cells per sample).

Interestingly, very high fractions of schizont irbcs could be obtained relying on a basic principle of HGMS: When performing HGMS in a column with ferromagnetic material as a matrix, any particle with a higher magnetic susceptibility will replace a particle with a lower magnetic susceptibility at the wire matrix of the column. Previously, Moore *et al *have demonstrated the continuously rising magnetic susceptibility of irbc with age [10]. In figure 2 eleven HGMS concentration experiments that were performed with non-synchronized cultures are analyzed by considering the *number of schizonts *irbc applied to the column (as opposed to considering the *total number *of irbcs). It can be seen that regularly, high purities of very late-stage, segmented irbc can be achieved by overloading the column with schizont irbc. In other words, when loading a non-synchronized culture containing equal or more than 5 × 10^7 ^schizont irbc onto the column, the schizont/total irbc fraction in the isolate reaches between 82% and 92%, while total irbc purities range between 94% and 98%. For example, this can be achieved by applying 250 μl packed cells from a regular, non-synchronized *Plasmodium *culture with a segemented-stage parasitemia of ≥ 2% onto a HGMS concentration column. The morphology of the isolate of a representative experiment is shown in figure 3.

**Figure 4:**
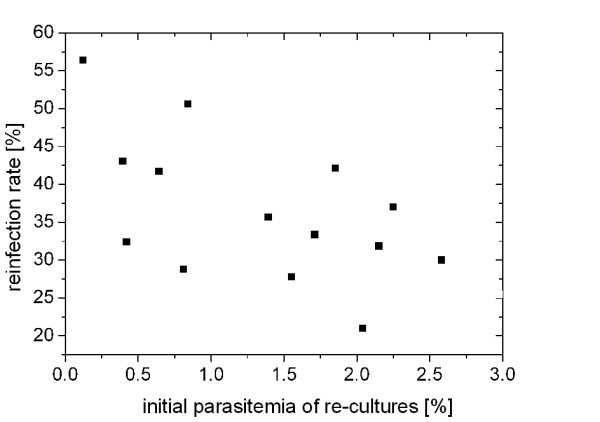
**Infection rate of re-cultured HGMS purified parasites**. Initial parasitemias of re-cultures were adjusted between 0.12% and 2.58%. Infection rate was calculated assuming 10 merozoites per schizont. The apparent influence of initial parasitemia on re-infection rate was not significant.

To examine viability of rbc and parasites exposed to the stainless steel matrix and the strong magnetic field during the HGMS, the infection rate upon re-culturing of HGMS-purified parasites was determined. In 14 experiments it ranged from 21.0% to 56.4% (mean: 36.57%, SD 9.46%), which confirmed satisfactory viability of re-cultured parasites (the starting parasitaemia of re-cultures was adjusted between 0.12% to 2.58%) (Figure 4).

### Depletion of trophozoites and schizonts from cultures by buffer-optimized HGMS

Five experiments comparing HGMS depletion and sorbitol lysis were performed and analyzed microscopically. All experiments were carried out with 50 μl packed cells from *Plasmodium *cultures. Complete depletion of schizonts and late trophozoites by HGMS depletion was observed up to a maximum column capacity of 23 million lates-stage irbc (Table 2). Quality of depleted cultures was determined by re-culturing as described below.

**Table 2:** HGMS-depletion experiments

	Initial culture:		HGMS depleted fraction:	
**Experiment No.**	**Rings+early trophozoites [%]**	**Late trophozoites + schizonts [%]**	**Rings+early trophozoites [%]**	**Late trophozoites + schizonts**

1	5.32	0.38	5.19	none

2	3.54	0.77	3.21	none

3	11.57	1.18	11.86	none

4	4.60	1.68	4.30	none

5	4.67	4.48	4.69	none

For each experiment, 50 μl of packed cells from Plasmodium culture were applied to the depletion column as described in material and methods. The flow-through was collected and analyzed microscopically (1000 rbc counted). Experiments were performed on different days from different cultures.

### Comparison of cultures after HGMS depletion and sorbitol lysis

After HGMS depletion or sorbitol lysis, for each method identical numbers of ring-stage irbc were re-cultured. Initial parasitaemia was adjusted to 0.5-1% with fresh rbc ("day 0"). For example, 500 million rbc with a differential parasitaemia of 4.0% ring and young trophozoite irbc and 4.6% late trophozoite and schizont irbc were processed by HGMS-depletion over one column or by sorbitol lysis. The flow-through or the remaning cells of sorbitol lysis, respectively, were washed and resuspended in complete culture medium. For one 25 ml cell culture flask, 250 million rbc with a total of 10 million ring-stage irbc were re-cultured by adding 75 μl of fresh rbc which resulted in a final ring-stage parasitaemia of 1.0%.

**Figure 5:**
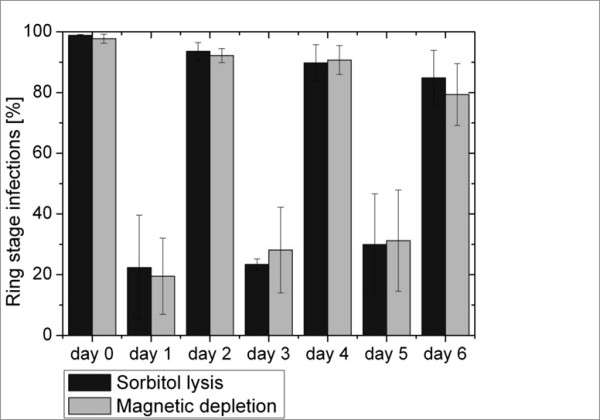
**Comparison of culture development after HGMS-depletion and sorbitol lysis**. Bars represent the fraction of ring-stage irbc out of total irbc. Percentage of ring-stage irbc decrease over time from 97.7% for HGMS depletion and 98.9% for sorbitol lysis on day 0 (day of experiment and re-culture) to 79.3% for HGMS depletion and 84.8% for sorbitol lysis on day 6. No significant difference was observed between both methods. Data are from 5 independent experiments for HGMS-depletion and from 3 independent experiments for sorbitol lysis.

To compare development of cultures after HGMS depletion and sorbitol lysis, differential parasitemias were counted by flow cytometry every 24 h at the same time of day, for six following days. Both methods resulted in cultures that developed in an essentially identical manner over the 6 following days (Figure 5).

## Discussion

Although irbc concentration by Percoll^® ^gradient centrifugation, gelatin sedimentation and depletion by sorbitol lysis have remained the gold standards for separation of late-stage irbc for the last decades, all three methods still harbour certain disadvantages. Separation results are not always consistent, depending on a variety of factors including the individual experience of the investigator, and gelatin sedimentation can be used only for parasite strains exhibiting knobs.

HGMS represents an alternative method for isolation of malaria irbc. In theory, irbc concentration as well as irbc depletion should be achievable. As to the latter, no reports are available. As to the first, after the initial report of Paul *et al *in 1981 [14], few other studies focussed on the subject. Employing commercially available polymer-coated HGMS columns (MACS^®^), Staalsohe *et al *[16] and Uhlemann *et al *[17], reported separation purities of irbc of 70-90%, with purities over 90% only achievable through the use of a second column. Data for maximum column capacity are not given. Another study describes successful concentration of trophozoits, schizonts and gametocytes from different malaria species, with purities up to 98% for *P. falciparum *[18]. Again, total column capacities are not documented. Purities of irbc of up to 98% were achieved using polymer coated HGMS columns for concentration of *Plasmodium berghei *irbc from mice [15]. A recently published report employed HGMS for synchronization of *P. falciparum *cultures. However, pre-treatment of cultures by sorbitol lysis was essential for synchronization, and overall purities of HGMS concentration were rather variable, ranging from 13.60% to 85.28% [19]. Other publications report the use of HGMS for purification of *Plasmodium *irbc, but rely on the protocols described in the studies mentioned above, and do not focus on the subject of HGMS as such.

This study presents a detailed report of new approaches to HGMS concentration and depletion, which were developed for malaria research.

For HGMS concentration, the introduction of density and viscosity optimized buffers renders polymer coating of the HGMS matrix unnecessary and regularly achieves irbc purities of over 95% from initial parasitaemias as low as 1.7%.

For HGMS depletion of late-stage irbc, the protocol offers a first viable alternative to sorbitol lysis for late-stage depletion of malaria cultures. For this, however, some limitations and handling challenges should be considered to help researchers decide under which circumstances it might be the method of choice:

First of all, capacities of columns are limited to a certain maximum number of cells. In our setting, a total of 22 million late-stage irbc was determined as capacity limit of the columns. In a conventional culture with about 1 to 2.5% late-stage parasitaemia and a total parasitaemia of 5%, this would correspond to loading roughly 10^9 ^total rbc (100 μl packed cells) onto the column, which in our laboratory is equivalent to a culture from a 25 ml flask. For this case, the processing time of 50 min is similar to sorbitol lysis. Upscaling of columns is under way and will be able to offer larger capacities in the future.

HGMS concentration and depletion can be combined and repeated, just as Percoll and sorbitol protocols, to achieve very highly synchronized malaria cultures. In principle, some concern might be raised from repeated exposure of rbc and parasites to the rather strong magnetic field and to possible contaminants derived from the stainless steel wool matrix. Even though effects cannot be ruled out with complete certainty, no impact on the viability and morphology of parasites was observed in this study. This is corroborated by a previous report that showed unchanged viability and morphology of erythrocytes, leukocytes and platelets after passage through an uncoated HGMS column [23]. Quite obviously, significant corrosion of high-grade stainless steels takes much longer periods of time than the exposure interval of cells to the column matrix in this study. However, caution might be recommended for studies examining the iron metabolism of parasites, since obviously, contamintation with iron from the matrix cannot be totally excluded. Nevertheless, in summary, the complete absence of synthetic chemicals and osmotically active substances is considered an advantage of buffer-optimized HGMS over conventional methods. A comparison between existing irbc concentration and depletion methods in malaria research is given in Table 3.

**Table 3:** Comparison between existing irbc concentration and depletion methods and buffer optimized HGMS

Method	Application	Purity	Assay time	Cost per sample^A^	Special equipment required
**Percoll^®^:**	*concentration*	50-99%	1-1.5 h	2-3 USD	none

**Sorbitol lysis:**	*depletion*	97-99%	50 min	1-2 USD	none

**Polymer-coated HGMS:**	*concentration*	83-99%	45 min	10-15 USD	Magnet, HGMS columns

	*depletion*	NA	NA	-	

**Buffer optimized HGMS:**	*concentration*	93-99%	45 min	6-8 USD	Magnet, HGMS columns
		
	*depletion*	97-98%	50 min		

^A ^For concentration methods cost was estimated for isolation of 10^7 ^irbc. assuming 95% purities of separations. Depletion cost refers to depletion of 2 × 10^7 ^irbc from cultures.

## Conclusion

Buffer-optimized HGMS has the potential to become a useful tool in malaria research. When compared to conventional methods and to commercially available HGMS systems, the high quality of irbc purifications, ease of handling and low cost should render buffer-optimized HGMS attractive for malaria researchers in a wide range of fields.

## List of abbreviations

AO: acridine orange; BSA: bovine serum albumin; HGMS: high gradient magnetic separation; HE: hydroethidine; irbc: infected red blood cells; rbc: red blood cells.

## Competing interests

SCB and PM are directors of a company developing HGMS systems for biomedical research. The other authors declare that they have no competing interests.

## Authors' contributions

SCB conceived, designed and tested the method, and drafted the manuscript. AO and SS participated in designing and performing experiments. PS, PP and PC analysed results of experiments. PM and KP revised the manuscript. HPN conceived the study and revised the manuscript. All authors read and approved the final manuscript.
